# Electronic States of Nanocrystal Doped with Oxygen and Visible Emission on Black Silicon Prepared by ns-Laser

**DOI:** 10.1186/s11671-017-2209-3

**Published:** 2017-07-12

**Authors:** Zhong-Mei Huang, Wei-Qi Huang, Shi-Rong Liu, Xue-Ke Wu, Chao-Jian Qin

**Affiliations:** 1 Institute of Nanophotonic Physics, Guizhou University, Guiyang, 550025 China; 2 Surface Physics Laboratory, Department of Physics, Fudan University, Shanghai, 200433 China; 3 State Key Laboratory of Environmental Geochemistry Institute of Geochemistry, Chinese Academy of Science Institute of Geochemistry, Guiyang, 550003 China

## Abstract

We fabricated the black silicon (BS) structures by using nanosecond pulsed laser (ns-laser) in vacuum or in oxygen environment. It is interesting that the enhanced visible emission occurs in the photoluminescence (PL) spectra measured at room temperature and at lower temperature on the BS surface after annealing, in which lasing near 600 nm is observed on the BS surface with Purcell cavity structure. It is demonstrated in the PL spectra analysis that the electronic states in the nanocrystal doped with oxygen play a main role in the visible emission on the BS surface. The origin of the visible emission near 400, 560, or 700 nm is univocally revealed in the PL spectra analysis. A visible emission is promising for the development of the white light device on the BS.

## Background

Bulk silicon has an indirect band gap of 1.12 eV and poor emission efficiency. However, scientists think that developing efficient silicon light emitter is crucial for integrating optoelectronic devices into silicon-based chip. Recent reports demonstrate that visible emission at room temperature occurs in low-dimensional nanostructures of silicon [1–6], especially in the black silicon (BS) structure fabricated by using pulsed laser [7–12]. A simple pulsed laser (femtosecond (fs) or nanosecond (ns) laser) processing technique can drastically change the optical properties on silicon. In particular, visible emission on the BS surface attracts scientific interest, where the emission mechanism is still under debate [13–15].

In the letter, we fabricated the BS surface structure by using ns-laser in vacuum or in oxygen environment, in which efficient emission in visible range was observed. It is interesting that the suitable annealing condition on the BS can obviously improve the visible emission owing to crystallizing process. More interesting, the visible emission measured at room temperature can be enhanced on the BS prepared in oxygen environment. The analysis of photoluminescence (PL) spectra and TEM image demonstrates that the Si nanocrystals (NCs) doped with oxygen play a main role in the visible emission on the BS, and the mechanism of visible emission near 420, 560, and 700 nm is univocally revealed. These observations imply the potential in fabricating silicon-based solid state lighting and light sources for visible range.

## Experiments and Results

**Fig. 1:**
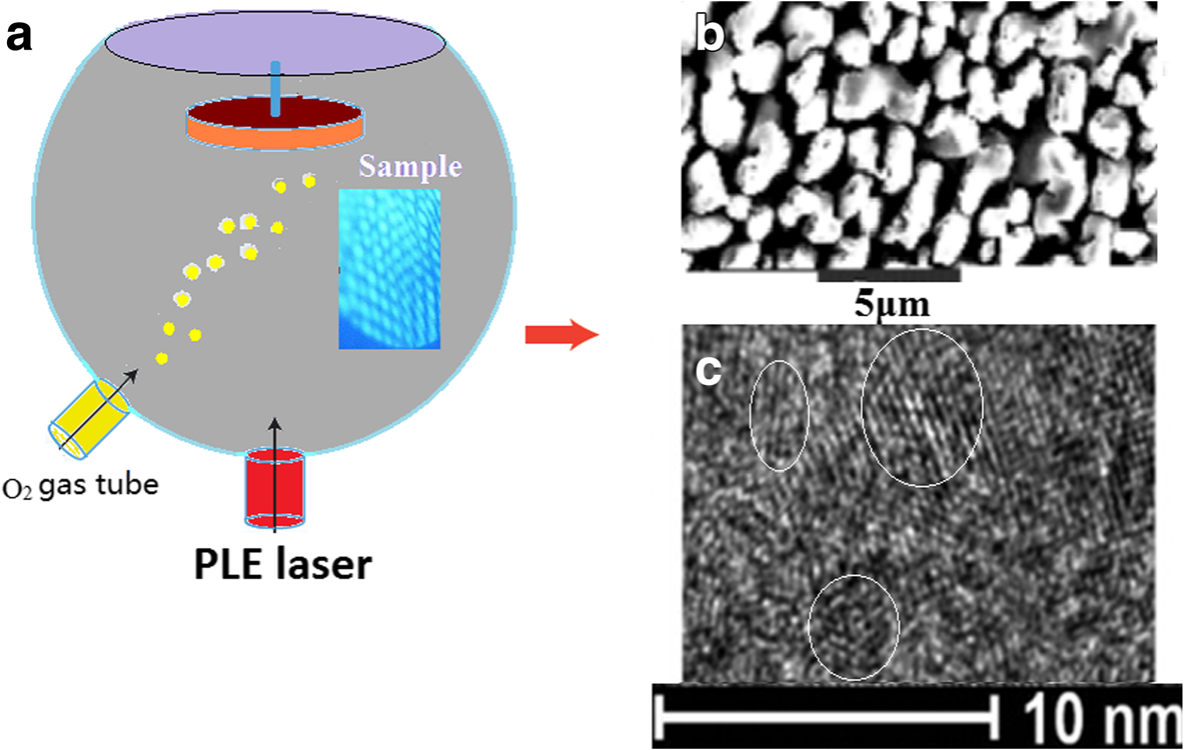
**a** Structure depiction of PLE device used to fabricate the BS structures. **b** SEM image of the BS surface structure prepared by ns-laser after annealing. **c** TEM image of nanosilicon in the BS prepared by ns-laser after annealing

A pulsed laser etching (PLE) device is used to fabricate the BS surface structures, in which the spot diameter of ns-laser is about 10 μm focused on the silicon wafers of P-type substrate with 10 Ωcm in vacuum (sample I) or in oxygen environment with 80 Pa (sample II), as shown in Fig. 1a. It is interesting that the plasmonic lattice structure occurs on the BS surface in PLE process as shown in the inset of Fig. 1a. SEM image in Fig. 1b shows the BS surface structure prepared by ns-laser after annealing, on which the reflective rate is lower than 10% and the refractive index is about 1.88 in visible range on the SiO_2_ surface. These experimental results agree with the K-K relations [16, 17]. The nanocrystals of silicon occur in the BS prepared by ns-laser after annealing, as shown in the TEM image of Fig. 1c.

The PL spectra on the samples are measured under the 266-nm excitation laser at room temperature (300 K) and lower temperature (10~200 K) in the sample chamber of 1 Pa.

It should be noted that the temperature and the time in annealing on the BS are important due to crystallizing process. The annealing at 1000 °C is suitable for visible emission in the PL spectra measured in 10 K on the BS prepared in vacuum (sample I), and the optimal annealing time is about 15 min at 1000 °C for visible emission in the PL spectra measured at room temperature on the BS prepared in oxygen of 80 Pa (sample II).

It is very interested to make a comparison between the sample I prepared in vacuum and the sample II prepared in oxygen with 80 Pa in the analysis of PL spectra at different temperature.

**Fig. 2:**
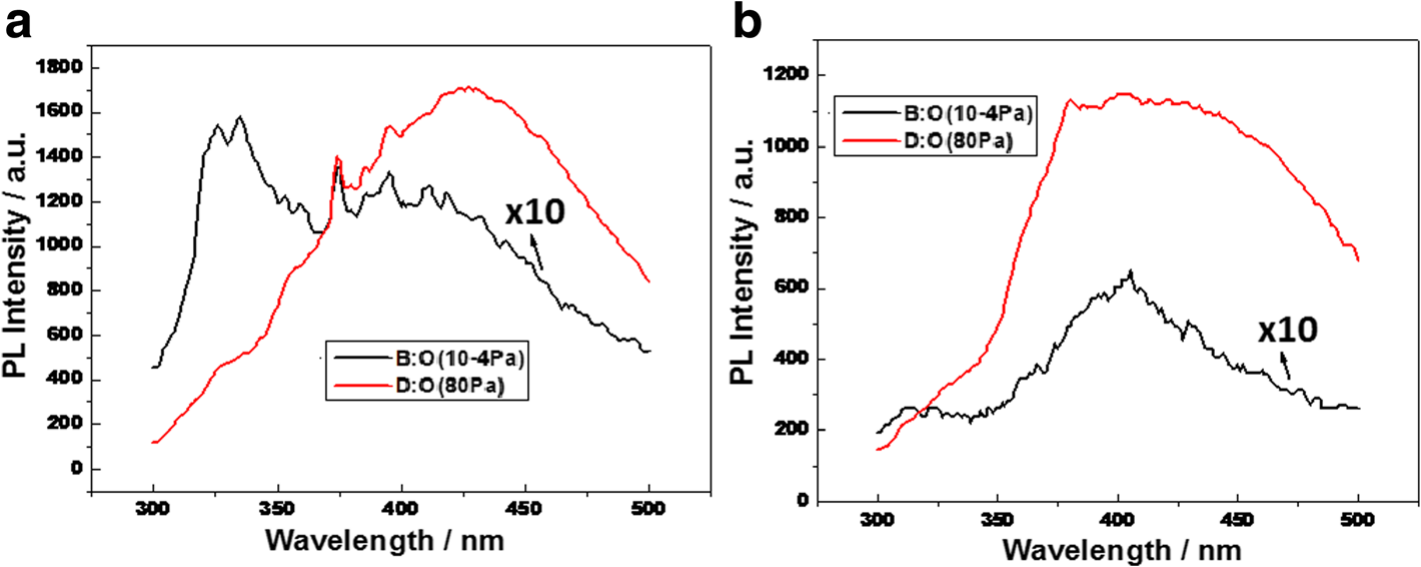
**a** PL spectra from 300 to 500 nm measured at lower temperature on the sample I (*black curve*) and the sample II (*red curve*). **b** PL spectra measured at room temperature on the sample I (*black curve*) and the sample II (*red curve*), in which the impurity states on nanocrystals are exhibited in the broader enhanced PL peaks on the sample II

It is detailedly exhibited that the peak intensity in shorter wavelength near 330 nm measured at 10 K on the sample I prepared in vacuum is stronger as shown along with the black curve in Fig. 2a which may be originated from the nanocrystal emission, but the PL intensity in longer wavelength near 400 nm measured at room temperature on the sample II prepared in oxygen with 80 Pa is obviously enhanced as shown along with the red curve in Fig. 2b.

**Fig. 3:**
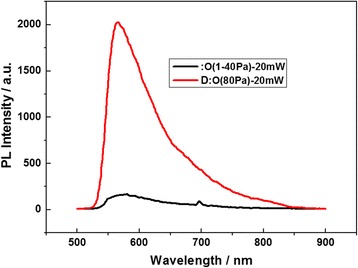
PL spectra near 560 nm measured at room temperature made a comparison between the sample I (*black curve*) and the sample II (*red curve*)

It is more interesting to make a comparison between the sample II and the sample I in PL spectra analysis near 560 nm. The PL peak measured near 560 nm at room temperature is enhanced on the BS sample II prepared in oxygen of 80 Pa as shown along with the red curve in Fig. 3 related to the impurity states on nanocrystals, while the PL intensity near 560 nm is weaker on the BS sample I prepared in vacuum as shown along with the black curve in Fig. 3.

**Fig. 4:**
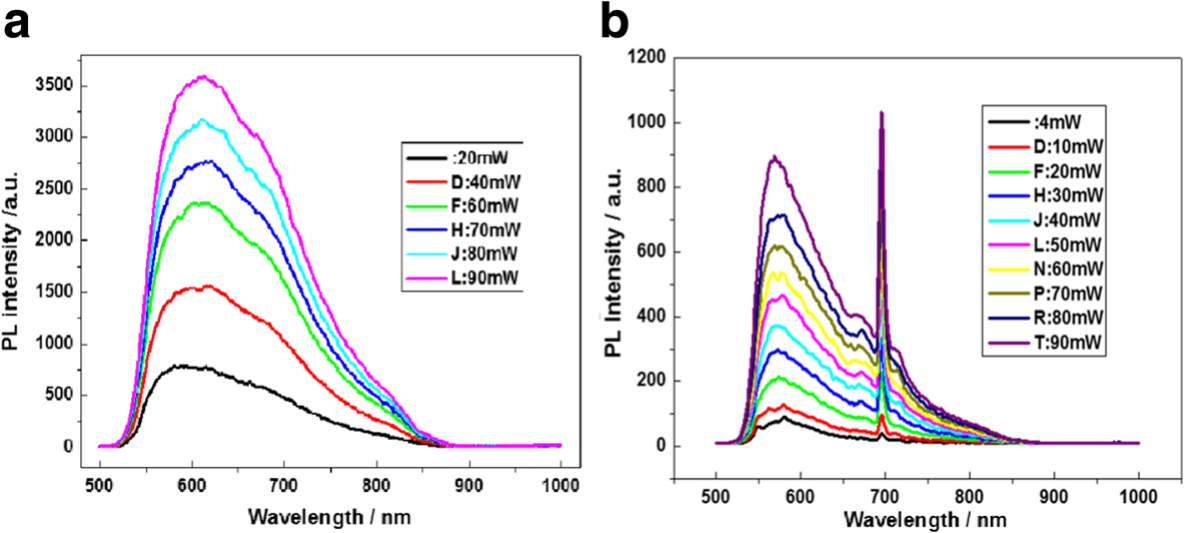
**a** PL spectra with excitation power measured at room temperature on the sample I prepared in vacuum. **b** PL spectra with excitation power measured at room temperature on the sample I after annealing

Figure 4a shows the PL spectra with excitation power measured at room temperature on the sample I prepared in vacuum, in which the broader PL band is originated from the size distribution of nanocrystals in the BS. The analysis of PL spectra demonstrates that the broader band emission originated from the size distribution of nanocrystals disappears obviously, while the impurity states emission occurs near 600 and 700 nm after annealing at 1000 °C, as shown in Fig. 4b.

**Fig. 5:**
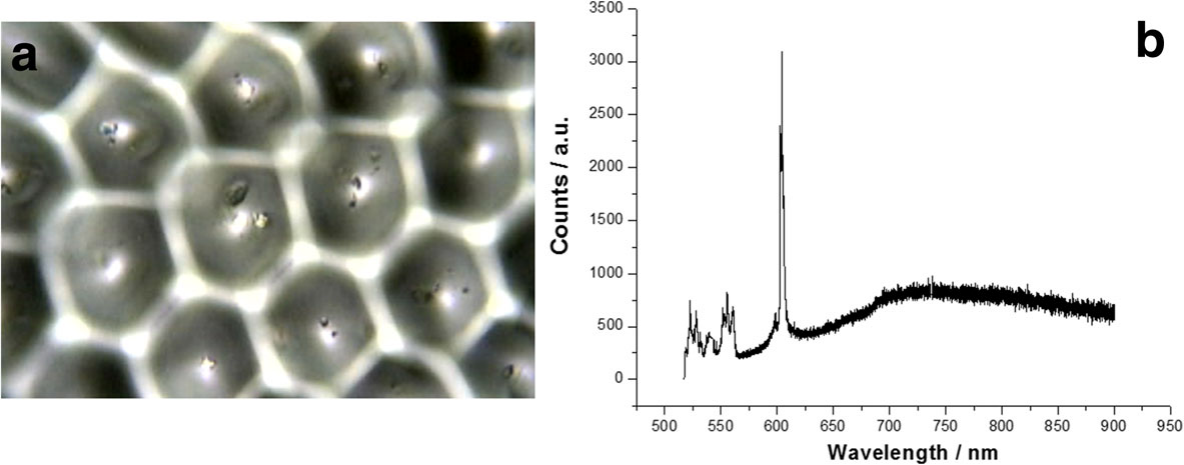
**a** Optical image of Purcell cavity structure in micrometer scale on the BS surface. **b** Sharper PL peak with lasing near 600 nm measured at room temperature on Purcell cavity structure in a micrometer scale on the BS surface under excitation laser at 514 nm

More interesting, the sharper PL peak with lasing near 600 nm occurs in Purcell cavity structure in micrometer scale on the BS under excitation laser at 514 nm, as shown in Fig. 5. Figure 5a shows the optical image of Purcell cavity structure in micrometer scale on the BS surface, and Fig. 5b shows the sharper PL peak with lasing near 600 nm on the BS after suitable annealing, in which the optical gain measured by using various strip length method is about 130 cm^−1^.

## Discussion

**Fig. 6:**
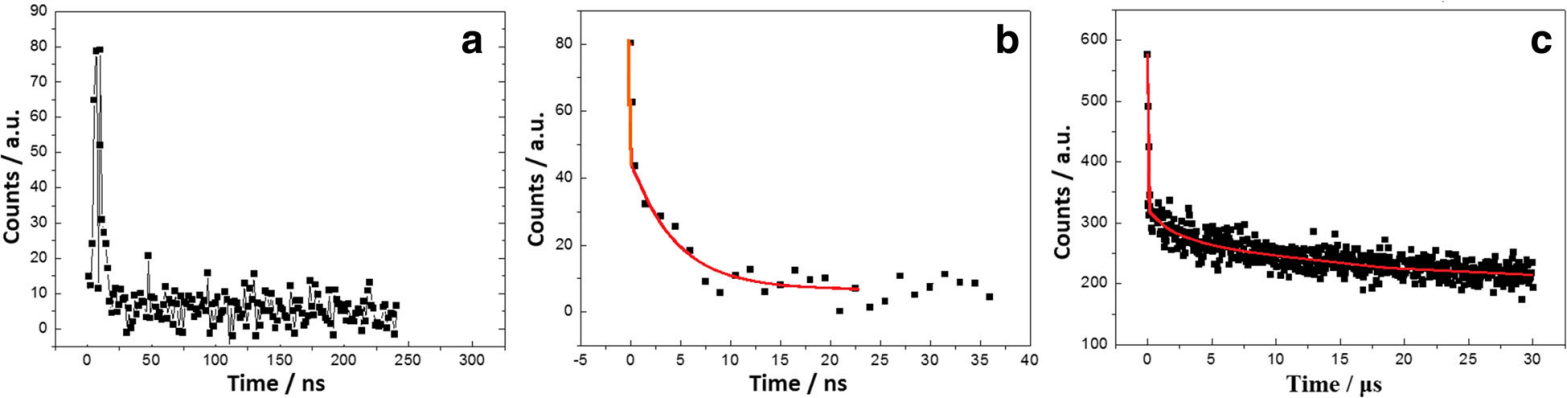
**a** PL decay spectra near 400 nm with the faster photons. **b** PL decay spectra near 560 nm with the faster photons (ns) on smaller Si NCs. **c** PL decay spectra near 700 nm with the faster photons (ns) related to the impurity state emission and the slower photons (μs) on larger Si NCs

The analysis of the PL decay spectra on Si NCs with various diameters demonstrates that the transformation from indirect gap to direct gap appears on the smaller Si NCs, as shown in Fig. 6a, b. The direct-gap emission near 400 and 560 nm relates to the faster photons on the smaller NCs (diameters <2 nm), and the indirect-gap emission relates to the slower photons (involving phonon assistance process) on the larger NCs (diameters >2.5 nm). Figure 6c shows the PL decay spectra near 700 nm involving the slower photons (~μs) on the larger NCs and the faster photons (~ns) owing to the impurity states.

**Fig. 7:**
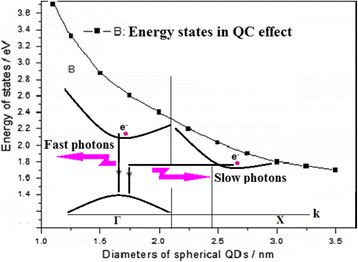
The emission model depiction from the analysis of the PL decay spectra on Si NCs with various diameters, in which the direct-gap emission relates to the faster photons on the smaller NCs (diameters <2 nm), and the indirect-gap emission relates to the slower photons (involving phonon assistance process) on the larger NCs (diameters >2.5 nm)

As shown in Fig. 7, in this emission model, the direct-gap emission relates to the faster photons on the smaller NCs (diameters <2 nm), and the indirect-gap emission relates to the slower photons (involving phonon assistance process) on the larger NCs (diameters >2.5 nm), which is along with the energy states’ curve in the quantum confinement effect.

## Conclusion

In conclusion, the microstructure and the nanostructure were found in the BS prepared by ns-laser. In the PL spectra on the BS surface structures, the emission peaks were measured in visible wavelength for LED application. We have compared the PL spectra on the BS samples I prepared in vacuum and the sample II prepared in oxygen of 80 Pa by ns-laser, in which it is demonstrated that the visible emission measured at room temperature near 400, 560, 600, and 700 nm is originated from the oxygen impurity states on the Si nanocrystals of the BS, while the emission near 330 nm measured at 10 K is owing to the nanocrystals emission. It is a new road to obtain emission devices for application of visible LED on silicon chip.

## Methods

### Photoluminescence Measurement

Photoluminescence (PL) spectra of the samples are measured under the 266 or 488 nm excitation at room temperature (300 K) and lower temperature (17~200 K) in sample chamber of 1 Pa. In the PL spectra, the sharper peaks with stimulated emission and direct-gap emission characteristics have been observed, in which the PL peak with lasing near 600 nm on the BS after suitable annealing is measured by using various strip length methods whose optical gain is about 130 cm^−1^. The PL decay spectra near 400, 560, and 700 nm are measured under ps-pulsed laser at 266 nm.
